# RPA-CRISPR/Cas12a mediated isothermal amplification for visual detection of *Phytophthora sojae*

**DOI:** 10.3389/fcimb.2023.1208837

**Published:** 2023-05-26

**Authors:** Yufang Guo, Hongming Xia, Tingting Dai, Tingli Liu

**Affiliations:** ^1^ Co-Innovation Center for the Sustainable Forestry in Southern China, Nanjing Forestry University, Nanjing, Jiangsu, China; ^2^ Jiangsu Provincial Key Construction Laboratory of Special Biomass Resource Utilization, Nanjing Xiaozhuang University, Nanjing, China

**Keywords:** *Phytophthora sojae*, CRISPR/Cas12a, recombinase polymerase amplification, disease diagnosis, detection

## Abstract

**Introduction:**

*Phytophthora sojae* is among the most devastating pathogens of soybean (Glycine max) and severely impacts soybean production in several countries. The resulting disease can be difficult to diagnose and other Phytophthora species can also infect soybean. Accurate diagnosis is important for management of the disease caused by *P. sojae*.

**Methods:**

In this study, recombinase polymerase amplification (RPA) in combination with the CRISPR/Cas12a system were used for detection of *P. sojae*. The assay was highly specific to *P. sojae*.

**Results:**

The test results were positive for 29 isolates of *P. sojae*, but negative for 64 isolates of 29 Phytophthora species, 7 Phytopythium and Pythium species, 32 fungal species, and 2 Bursaphelenchus species. The method was highly sensitive, detecting as little as 10 pg.µL^−1^ of *P. sojae* genomic DNA at 37°C in 20 min. The test results were visible under UV light and readout coming from fluorophores. In addition, *P. sojae* was detected from natural inoculated hypocotyls of soybean seedlings using this novel assay. The rapidity and accuracy of the method were verified using 30 soybean rhizosphere samples.

**Discussion:**

In conclusion, the RPA-CRISPR/Cas12a detection assay developed here is sensitive, efficient, and convenient, and has potential for further development as a kit for monitoring root rot of soybean in the field.

## Introduction

*Phytophthora sojae* is among the most destructive pathogens of soybean (*Glycine max*). An estimated annual worldwide loss of US$1–2 billion is caused by *P. sojae*, which was first identified as a soybean pathogen in the 1950s in Indiana, Ohio, and North Carolina (Schmitthenner, 1985; Tyler, 2007; Dorrance, 2018). The pathogen has gradually spread to many soybean-producing regions of the world, including Brazil, Canada, China, and Argentina, where it has become a major impediment to soybean production (Wrather and Koenning, 2006).

China is the third-largest producer of soybeans in the world, following the United States and Brazil (Zhang et al., 2010; Roese and Pereira Goulart, 2013). *P. sojae* was first detected in Heilongjiang Province of China in 1989 (Su and Shen, 1993; Cui et al., 2010). After assessing its potential risk to economic and agricultural security, the Ministry of Agriculture of the People’s Republic of China listed *P. sojae* as a quarantine pathogen in 2007. The disease has caused severe soybean losses in China and has shown a trend for gradual expansion in the country (Chen et al., 2004).

Rapid detection of *P. sojae* is a crucial step towards effective control of soybean root rot and seedling blight. Traditionally, methods to identify *P. sojae* involve direct isolation from diseased plant tissues and baiting from soil on semi-selective media (Davison, 1998). Subsequent identification based on pathogen morphology and DNA sequences are usually time-consuming and requires trained personnel. The development of a PCR-based assay for rapid and sensitive detection of *P. sojae* may facilitate efficient pathogen identification and lead to effective disease management. A variety of molecular detection methods for phytopathogens have been developed, including PCR (Wang et al., 2006; Bienapfl et al., 2011; Xiong et al., 2019), real-time fluorescent PCR, loop-mediated isothermal amplification (LAMP), and recombinase polymerase amplification–lateral flow dipstick (RPA-LFD) assays (Cullen et al., 2001; Cullen et al., 2002; Lees et al., 2002; Dai et al., 2012; Dai et al., 2015; Rojas et al., 2017; Dai et al., 2019). However, these detection procedures rely on thermal-cycling instruments and other specialized equipment for sample treatment and result output, which limits the application of these methods to diagnose diseases in the field and by gel electrophoresis (Cullen et al., 2001; Cullen et al., 2002; Lees et al., 2002; Filion et al., 2003; Zhu et al., 2021).

In recent years, several molecular detection technologies based on isothermal amplification reactions have been developed and applied to pathogen detection, such as nuclear acid sequence-based amplification (NASBA), rolling circle amplification (RCA), helicase-dependent amplification (HAD), LAMP, and recombinase polymerase amplification (RPA) (Polstra et al., 2002; Andresen et al., 2009; Clancy et al., 2015; Magriñá Lobato and O’Sullivan, 2018; Huang et al., 2019; Zhang et al., 2020; Xu Z. C. et al., 2022). In 2018, a new DNA detection method [DNA endonuclease targeted CRISPR trans reporter (DETECTR)] by combining CRISPR/Cas with isothermal amplification was developed (Chen et al., 2018). DETECTR uses RPA to amplify targeted double-stranded DNA (dsDNA) at a constant temperature. When Cas12a specifically recognises and cleaves the amplified product under the control of a programmable specific CRISPR RNA(crRNA), non-specific trans-cleavage activity is activated, which cleaves the single-stranded DNA (ssDNA) reporter in the system, generating a fluorescence signal. DETECTR is used for the rapid detection of viruses, and the sensitivity can reach the attomole level (Barrangou and Marraffini, 2014; Le et al., 2021; Wang et al., 2021; Yue et al., 2021).

Because the adjustable properties of CRISPR-Cas effectors, such as simplicity of design, easy operation, collateral cleavage activity, and high biocompatibility, CRISPR-Cas systems have been widely used in genome editing and transcriptional regulation (Kadam et al., 2023). The first biological evidence that CRISPR-Cas systems play a role in adaptive immunity was reported in 2007 when *S. thermophilus* CRISPR loci were shown to acquire novel spacers derived from the invasive phage DNA (Barrangou et al., 2007). In recent years, technologies based on CRISPR/Cas12a and Cas13a collateral cleavage activity have been used to detect COVID-19, nCOV-2019, African swine fever virus (ASFV), Zika virus (ZIKV), Dengue virus (DENV), and other important viruses (Myhrvold et al., 2018; Bai et al., 2019; Broughton et al., 2020). In addition, this novel technique can be used for rapid detection of *Salmonella*, *Brucella*, *Helicobacter*, and other bacteria (Dai et al., 2022; Liu et al., 2022; Xu J. H. et al., 2022). To date, the method has been used to detect not only bacteria, viruses, and mycoplasmas, but also phytopathogenic fungi causing diseases such as citus black star disease, citrus scab, and wheat blast (Shi, 2021; Shin et al., 2021; Li et al., 2022).

In this study, a novel RPA-CRISPR/Cas12a assay was developed for detection of *P. sojae*, targeting the *Ypt1* gene, comprising 10 min for crude DNA extraction, 5 min for the RPA reaction, and 15 min for the CRISPR/Cas12a reaction. The results can be observed as green fluorescence under a blue LED transilluminator at a wavelength of 470 nm or detected using a multifunctional microplate reader (fluorescence excitation wavelength [λ_ex_] 485 nm, fluorescence emission wavelength [λ_em_] 520 nm). The specificity of the assay was evaluated by testing against *P. sansomeana*, *P. melonis*, *P. vignae*, and other oomycete and fungal species. The analytical sensitivity and feasibility of this method were confirmed using artificially inoculated samples.

## Materials and methods

### Maintenance of isolates and DNA extraction

Twenty-nine isolates of *Phytophthora sojae* were recovered from roots, stems, and rhizosphere soil of diseased soybean plants in the Jiangsu, Anhui, and Heilongjiang provinces in China from 2002 to 2022 (**Table 1**). Sixty-four isolates of 29 *Phytophthora* species, 7 isolates of five species of other oomycetes, 32 isolates of 31 fungal species, and 2 isolates of *Bursaphelenchus* species used in this study were obtained from a collection maintained at the Department of Plant Pathology of Nanjing Forestry University (Nanjing, China) (**Table 1**). *Phytophthora sojae* and other oomycete isolates were grown on 10% clarified V8 juice agar in a 70-mm Petri dish and maintained in the dark at 20–25°C. After culture for 3–5 days, the mycelium was scraped from the medium surface. The fungal isolates were grown on potato glucose agar in 90-mm Petri dishes and maintained at 20°C in the dark. Genomic DNA (gDNA) was extracted from all isolates using the DNAsecure Plant Kit (Tiangen Biotech, Beijing, China) and quantified using a Nanodrop ND-1000 spectrophotometer (NanoDrop Technologies, Wilmington, DE, USA). All gDNA samples were stored at-20°C until use.

**Table 1:** Information and Crisp-cas12a deteciton results of *Phytophthora* and other oomycete and fungal isolates used in this study.

Number	(sub) clade	*Species*	Isolate	Origin		Crisp-cas12a deteciton results
				Host/substrate	Source	
1	7b	*Phytophthora sojae (R2)*	R2	*Glycine max*	B. M. Tyler	*+*
2		*P. sojae (R3)*	R3	*Glycine max*	J. H. Peng	*+*
3		*P. sojae (R6)*	R6	*Glycine max*	J. H. Peng	*+*
4		*P. sojae (R8)*	R8	*Glycine max*	J. H. Peng	*+*
5		*P. sojae (R12)*	R12	*Glycine max*	J. H. Peng	*+*
6		*P. sojae (R14)*	R14	*Glycine max*	J. H. Peng	*+*
7		*P. sojae (R17)*	R17	*Glycine max*	J. H. Peng	*+*
8		*P. sojae (R19)*	R19	*Glycine max*	B. M. Tyler	*+*
9		*P. sojae (R20)*	R20	*Glycine max*	J. H. Peng	*+*
10		*P. sojae (R28)*	R28	*Glycine max*	J. H. Peng	*+*
11		*P. sojae (R31)*	R31	*Glycine max*	J. H. Peng	*+*
12		*P. sojae *	Ps1	*Glycine max*	JS, China	*+*
13		*P. sojae *	Ps2	*Glycine max*	JS, China	*+*
14		*P. sojae *	Ps3	*Glycine max*	JS, China	*+*
15		*P. sojae *	Ps4	*Glycine max*	JS, China	*+*
16		*P. sojae *	Ps5	*Glycine max*	JS, China	*+*
17		*P. sojae *	Psf1	*Glycine max*	FJ, China	*+*
18		*P. sojae *	Psf2	*Glycine max*	FJ, China	*+*
19		*P. sojae*	Psf3	*Glycine max*	FJ, China	*+*
20		*P. sojae *	Psf4	*Glycine max*	FJ, China	*+*
21		*P. sojae *	Psf5	*Glycine max*	FJ, China	*+*
22		*P. sojae*	Psy1	*Glycine max*	YN, China	*+*
23		*P. sojae *	Psy2	*Glycine max*	YN, China	*+*
24		*P. sojae *	Psy3	*Glycine max*	YN, China	*+*
25		*P. sojae *	Psy4	*Glycine max*	YN, China	*+*
26		*P. sojae *	Psy5	*Glycine max*	YN, China	*+*
27		*P. sojae *	Psy6	*Glycine max*	YN, China	*+*
28		*P. sojae*	Psy7	*Glycine max*	YN, China	*+*
29		*P. sojae *	Psy8	*Glycine max*	YN, China	*+*
30		*P. vignae*	CPHST BL 30	*Vigna* sp.	M. D. Coffey	-
31		*P. melonis*	PMNJHG1	*Cucumis sativus*	JS, China	-
32		*P. melonis*	PMNJHG2	*Cucumis sativus*	JS, China	-
33		*P. melonis*	PMNJHG3	*Cucumis sativus*	JS, China	-
34		*P. melonis*	PMNJDG1	*Benincasa hispida*	JS, China	-
35		*P. melonis*	PMNJDG2	*Benincasa hispida*	JS, China	-
36		*P. melonis*	PMNJDG3	*Benincasa hispida*	JS, China	-
37	7a	*P. fragariae*	CBS209.46	*Fragaria × ananassa*	England, UK	-
38		*P. rubi*	CBS 967.95	*Rubus idaeus*	Scotland, UK	-
39		*P. cambivora *	CBS 248.60	*Castanea sativa*	USA	**−**
40		*P. cambivora *	Pc1	*Malus domestica* Borkh	SH, China	**−**
41	7c	*P. parvispora*	CBS132771	*Arbutus unedo*	Italy	-
42		*P. parvispora*	CBS132772	*Arbutus unedo*	Italy	-
43		*P.cinnamomi*	Pci1	*Pinus* sp.	AH,China	-
44		*P.cinnamomi*	Pci2	*Rhododendron simsii*	JS, China	-
45		*P.cinnamomi*	Pci3	*Cedrus deodara*	JS, China	-
46		*P.cinnamomi*	Pci4	*Camellia oleifera* Abel.	JS, China	-
47		*P.cinnamomi*	Pci5	*Pinus* sp.	JS, China	-
48		*P.cinnamomi*	Pci6	*Rhododendron simsii*	AH,China	-
49		*P.cinnamomi*	Pci7	*Rhododendron simsii*	SD, China	-
50		*P.cinnamomi*	Pci8	*Cedrus deodara*	SD, China	-
51		*P.cinnamomi*	Pci9	*Cedrus deodara*	AH,China	-
52		*P.cinnamomi*	Pci10	*Pinus* sp.	SD, China	-
53	*1*	*P. citricola*	Pcit	*Rhododendron pulchrum*	JS, China	**−**
54		*P. cactorum*	C1	*Malus pumila*	JS, China	-
55		*P. cactorum*	C2	*Malus pumila*	JS, China	-
56		*P. cactorum*	C3	*Rosa chinensis*	JS, China	-
57		*P. infestans*	Pi1	*Solanum tuberosum*	FJ, China	-
58		*P. infestans*	Pi2	*Solanum tuberosum*	YN, China	-
59		*P. nicotianae*	Pn1	*Nicotiana tabacum*	FJ, China	-
60		*P. nicotianae*	Pn2	*Lycopersicum* sp.	JS, China	-
61		*P. nicotianae*	Pn3	*Sophora sinensis*	JS, China	-
62		*P. nicotianae*	Pn4	*Citrus* sp.	JS, China	-
63		*P.tentaculata*	*Pt1*	*Aucklandia lappa*	YN, China	-
65	*2*	*P. pini*	*Ppini1*	*Rhododendron pulchrum*	*JS, China*	*-*
66		*P. pini*	*Ppini2*	*R. pulchrum*	*JS, China*	*-*
70		*P. capsici*	*Pc1*	*Capsicum annuum*	*JS, China*	*-*
71		*P. capsici*	*Pc2*	*Capsicum annuum*	*YN, China*	*-*
72		*P. capsici*	*Pc3*	*Capsicum annuum*	*SH, China*	*-*
73		*P.colocasiae*	*Pcol1*	*Colocasia esculenta (L.) Schott*	*YN,China*	*-*
74		*P.citricola*	*Pcitr1*	*Persea americana*	*JS, China*	*-*
75		*P. plurivora*	*Pplu1*	*Manihot esculenta*	*HN,China*	*-*
76	3	*P. ilicis*	CBS114348	*Ilex aquifolium*	Netherlands	-
77	4	*P. palmivora*	*Pp1*	*Iridaceae*	YN, China	-
78		*P. quercetorum*	15C7	*Soil*	USA	-
79	5	*P. castaneae*	CBS587.85	*Soil*	Taiwan	-
80	6	*P. megasperma*	CBS305.36	*Matthiola incana*	USA	-
81		*P. mississippiae*	57J3	*Irrigation water*	Mississippi, USA	-
82	8	*P. drechsleri*	CBS 292.35^T^	*Beta vulgaris* var*. altissima*	California (CA), USA	-
83		*P. drechsleri*	ATCC 56353	*Citrus sinensis*	Australia	-
84		*P. hibernalis*	CBS 270.31	*Cirrus sinensis*	USA	-
85		*P. syringae*	ATCC 34002	*Citrus* sp.	CA	-
86		*P. lateralis*	CBS168.42	*Cedrus deodara*	Canada	**−**
87		*P. ramorum*	EU1 2275	*Quercus palustris*	United Kingdom	-
88		*P. medicaginis*	ATCC 44390	*Medicago sativa*	USA	**−**
89	10	*P. boehmeriae*	Pb1	*Boehmeria nivea*	JS, China	-
90		*P. boehmeriae*	Pb2	*Gossypium* sp.	JS, China	-
91		*P. boehmeriae*	Pb3	*B. nivea*	JS, China	-
92		*P. boehmeriae*	Pb4	*Gossypium* sp.	JS, China	-
93	12	*P. quercina*	CBS 789.95	*Quercus petraea*	Australia	**−**
94	Oomycete	*Phytopythium litorale*	PC-dj1	*Rhododendron simsii*	JS, China	**−**
95		*P. helicoides*	PH-C	*Rhododendron simsii*	JS, China	**−**
96		*P. helicoides*	PF-he2	*Photinia × fraseri* Dress	JS, China	**−**
97		*P. helicoides*	PF-he3	*Photinia × fraseri* Dress	JS, China	**−**
98		*Pythium ultimum*	Pul1	*Citrus sinensis*	JS, China	**−**
99		*Py. spinosum*	Psp1	*Oryza sativa* L.	JS, China	**−**
100		*Py. aphanidermatum*	Pap1	*Nicotiana tabacum*	JS, China	**−**
102	Fungi	*Fusarium oxysporium*	Fox1	*Gossypium* sp.	JS, China	**−**
103		*F. solani*	Fso1	*Gossypium* sp.	JS, China	**−**
104		*F. solani*	Fso2	*Glycine max*	JS, China	**−**
105		*F. circinatum*	A045-1	*Pinus sp.*	SH, China	**−**
106		*F. fujikuroi*	Ffu1	*Oryza sativa*	JS, China	**−**
107		*F. graminearum*	Fgr1	*Triticum aestivum*	JS, China	**−**
108		*F. acuminatum*	Fac1	*Rhizophora apiculata*	SC, China	**−**
109		*F. asiaticum*	Fas1	*Triticum aestivum*	JS, China	**−**
110		*F. avenaceum*	Fav1	*Glycine max*	JS, China	**−**
111		*F. culmorum*	Fcu1	*Glycine max*	SC, China	**−**
112		*F. commune*	Fco1	Soil	HLJ, China	**−**
113		*F. equiseti*	Feq1	*Glycine max*	JS, China	**−**
114		*F. lateritium*	Flat1	Soil	JS, China	**−**
115		*F. moniforme*	Fmo1	*Oryza sativa*	JS, China	**−**
116		*F. nivale*	Fniv	*Triticum aestivum*	JS, China	**−**
117		*F. proliferatum*	Fpr1	*Pinus sp.*	JS, China	**−**
118		*F. incarnatum*	IL3HQ	*Medicago sativa*	JS, China	**−**
119		*Colletotrichum truncatum*	Ctr1	*Glycine max*	JS, China	**−**
120		*C. glycines*	Cgl1	*Glycine max*	JS, China	**−**
121		*C. orbiculare*	Cor1	*Citrullus lanatus*	JS, China	**−**
122		*Verticilium dahlia*e	Vda1	*Gossypium* sp.	JS, China	**−**
123		*Rhizoctonia solani*	Rso1	*Gossypium* sp.	JS, China	**−**
124		*Magnaporthe grisea*	Guy11	*Oryza sativa*	Japan	**−**
125		*Endothia parasitica*	Epa1	*Castanea mollissima*	JS, China	**−**
126		*Bremia lactucae*	Bla1	*Lactuca sativa*	JS, China	**−**
127		*Aspergillus flavus*	NJC03	*Actinidia chinensis*	SX, China	**−**
128		*Botrytis cinerea*	Bci1	*Cucumis sativus*	JS, China	**−**
129		*Alternaria alternata*	Aal1	Soil	JS, China	**−**
130		*Tilletia indica*	Tin1	*Triticum aestivum*	JS, China	**−**
131		*Diaporthe mahothocarpus*	DT1	*Kerria japonica*	JS, China	**−**
132		*D. sapindicola*	WHZ3	*Sapindus mukorossi*	JS, China	**−**
133		*Botryosphaeria dothidea*	Bci1	*Koelreuteria paniculata*	JS, China	**−**
134	Nematode	*Bursaphelenchus xylophilus*	Js-1	*Pinus thunbergii*	JS, China	**−**
135		*B.mucronatus*	Bmucro	*Pinus* sp.	JS, China	**−**

### Design of RPA primers, crRNA, and ssDNA reporter

The *Ypt1* gene was selected as the target for the design of gene-specific RPA primers (Dai et al., 2019). The RPA primers were designed using Primer Premier 6.0 (Premier Biosoft, Palo Alto, CA, USA) and based on the recommendations in the DNA Amplification Kit manual which were listed in **Supplementary Figure S1**. The CHOPCHOP web tool (http://chopchop.cbu.uib.no/) was used to design the CRISPR RNA (crRNA) ssDNA reporters (Zhao et al., 2022). The crRNA sequence did not overlap with that of the RPA primers and target conserved regions of the RPA amplicon (crRNA: UAAUUUCUACUAAGUGUAGAUCGAUCCAGUUGCAGUUGCUGACAAUA) (**Figure S1**). The 5′-end of the ssDNA reporter was tagged with 6-FAM and the 3′-end was tagged with the BHQ-1 quencher (5′ 6-FAM-TTATT-BHQ-1 3′) (Chen et al., 2018; Li et al., 2018). The crRNA and ssDNA reporter were synthesized by GenScript (Nanjing, China) and stored at -80°C until use.

### RPA-CRISPR/Cas12a assay

The 30-min assay included a two-step approach (5 min for the RPA reaction and 15 min for the CRISPR/Cas12a assay). First, the pair of RPA primers (Ypt1RPA-F/Ypt1RPA-R) were used to amplify the *Ypt1* gene of *P. sojae* in a 5-min RPA step. Second, the CRISPR/Cas12a system was used to detect and visualize the amplified products within 15 min.

**Figure 1:**
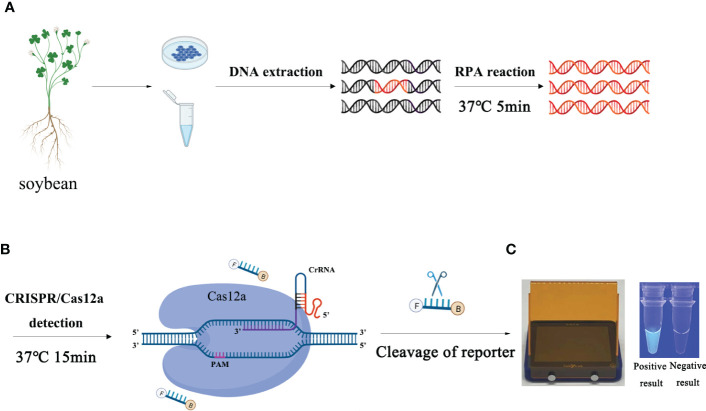
Schematic diagram of the RPA-CRISPR/Cas12a assay for detection of *Phytophthora sojae*. **(A)** Recombinase polymerase amplification. **(B)** Cas12a protein can combine with each amplicon and target-specific crRNA to form a complex with indiscriminate ssDNA cleavage activity. The FAM-labeled ssDNA reporter is cleaved and produces visible green fluorescence under excitation at a wavelength of 470 nm. **(C)** Positive result: visible green fluorescence. Negative result: no green fluorescence visible.

The detection process involved in the RPA-CRISPR/Cas12a analysis is shown in **Figure 1**. The RPA assay was performed in a 50-µL reaction mixture using the Test Strip Kit (LeShang Ltd., WuXi, China) in accordance with the manufacturer’s instructions. Each reaction mixture initially contained 2 µL of each forward and reverse primer (Ypt1RPA-F/Ypt1RPA-R, 10 µM), 25 µL rehydration buffer, 2 µL gDNA (10 ng.µL^−1^), and 16 µL double-distilled H_2_O (ddH_2_O), amounting to 47 µL in volume. After centrifugation of this mixture at 4000 rpm for 5 s, 3 μL of activator (supplied with the kit) was added to the lid of the reaction unit. The reaction unit was closed tightly, centrifuged at 4,000 rpm for 5 s, and then shaken manually for 3 s so that the mixture and activator were well mixed. Reactions were conducted at 37°C. After 4 min, the reactions were shaken manually and centrifuged at 4000 rpm for 5 s. The reaction tube was incubated at 37°C for 20 min. The RPA products were then analyzed using the CRISPR/Cas12a system.

The ssDNA reporter labeled with 6-FAM and BHQ-1 was added to the reaction tube of the CRISPR/Cas12a system. The Cas12a/crRNA recognized the target protospacer-adjacent motif sequence, the cleavage activity of Cas12a was generated, and the ssDNA reporter was cleaved, resulting in fluorescence. Various concentrations of crRNA (40 nM, 300 nM, 0.5 µM, 1 µM, 2 µM, 5 µM, and 10 µM) and ssDNA reporter (40 nM, 300 nM, 1 µM, 2 µM, 5 µM, and 10 µM) were used to screen the optimal concentration combination (**Table S1**). The optimal incubation time of the RPA reaction and the cleavage time of Cas12a were tested separately with eight time points (5, 10, 15, 20, 25, 30, 35, and 40 min). The CRISPR/Cas12a reaction was performed in a 50-μL reaction mixture, comprising 38 μL ddH_2_O, 5 μL reaction buffer, 3 μL crRNA, 1 μL Cas12a, 1 μL ssDNA reporter, and 2 μL RPA product. The reaction mixture was centrifuged at 4000 rpm for 5 s, then incubated at 37°C. The results of the RPA-CRISPR/Cas12a assay can be detected by two methods. Strong fluorescence signals were detected by a multifunctional microplate reader (λ_ex_ 485 nm, λ_em_ 520 nm) or visible green fluorescence was detected under a blue LED transilluminator at 470 nm wavelength, whereas no fluorescent signal or visible green fluorescence was detected in the negative controls. All RPA-CRISPR/Cas12a reactions were repeated at least three times. The STDEVP function was used to analyze the three results (number 1, number 2, and number 3) obtained by repeating CRISPR/Cas12a analysis three times to calculate the standard deviation. Statistical analysis was performed using GraphPad Prism 8 software (GraphPad Software Inc., San Diego, CA, USA). The experimental group and control group were compared by performing the Student’s t-test for a difference analysis by calculating P value. P <0.05 (*) was considered statistically significan.

### Specificity and sensitivity of the RPA-CRISPR/Cas12a assay

Specificity of the RPA-CRISPR/Cas12a assay was evaluated against all isolates (22 isolates of *P.sojae*, 64 isolates of 29 other *Phytophthora* species, 7 isolates of other oomycetes, 32 isolates of fungal species, and 2 isolates of *Bursaphelenchus* species) listed in **Table 1**. Purified gDNA samples (100 ng) were used as templates and a positive control (100 ng *P. sojae* isolate) and double-distilled H_2_O as a no template control (NTC) were included in each set of reactions. To determine sensitivity, 10-fold dilutions of *P. sojae* gDNA (isolate P6497) ranging from 100 to 0.0001 ng.µL^−1^ were used as DNA templates in the RPA-CRISPR/Cas12a assay. A negative control (NC; ddH_2_O) was included in each set of reactions. All RPA-CRISPR/Cas12a reactions were repeated at least three times. The STDEVP function was used to analyze the three results (number 1, number 2, and number 3) obtained by repeating CRISPR/Cas12a analysis three times to calculate the standard deviation. Statistical analysis was performed using GraphPad Prism 8 software (GraphPad Software Inc., San Diego, CA, USA). The experimental group and control group were compared by performing the Student’s t-test for a difference analysis by calculating P value. P <0.05 (*) was considered statistically significan.

### Detection of *P. sojae* in naturally infected soybean plants using the RPA-CRISPR/Cas12a assay

After inoculation of *P. sojae*, soybean plants seedlings had severe wilting with discoloration. Using a five-point sampling method, five soybean plants were collected at the booting stage at each sampling point from a field infected with soybean root rot. For sample processing, each of the ground tissues of 25 soybean plants were cut into 2-cm-long segments, and 250 g fresh samples were taken from each plant. The samples were ground for 1 min with a grinder (XB-M101, Lingrui, China). A sample (1 g) was placed in a 1.5-mL centrifuge tube containing 1 mL PEG-OH extraction buffer (1 g NaOH was dissolved in 900 mL ddH_2_O, 60 mL PEG200 was added, and the volume adjusted to 1 L with ddH_2_O, and sterilized with a bacterial filter) and incubated at room temperature (25°C) for 8 min. The supernatant was collected as the template for the RPA-CRISPR/Cas12a assay. Purified gDNA (10 ng.µL^−1^) extracted from healthy soybean plants and ddH_2_O were included in each repeat as a NC and NTC, respectively. The STDEVP function was used to analyze the three results (number 1, number 2, and number 3) obtained by repeating CRISPR/Cas12a analysis three times to calculate the standard deviation. Statistical analysis was performed using GraphPad Prism 8 software (GraphPad Software Inc., San Diego, CA, USA). The experimental group and control group were compared by performing the Student’s t-test for a difference analysis by calculating P value. P <0.05 (*) was considered statistically significan.

## Results

### Optimization of the RPA-CRISPR/Cas12a assay for detection of *P. sojae*

**Figure 2:**
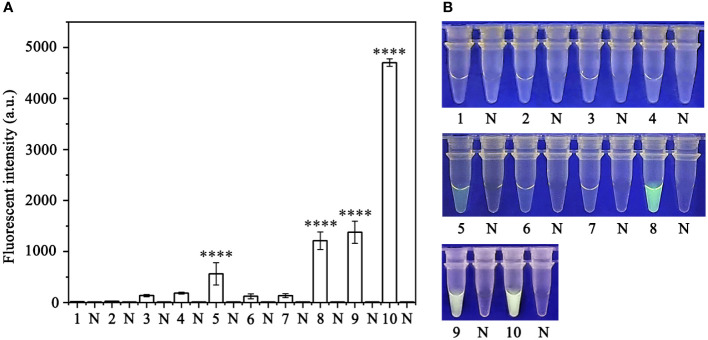
Optimization of the crRNA and ssDNA reporter concentrations for the RPA-CRISPR/Cas12a assay. The crRNA and ssDNA reporter concentrations were set to the following values: 1: 40 nM crRNA, 40 nM ssDNA reporter; 2: 300 nM crRNA, 40 nM ssDNA reporter; 3: 500 nM crRNA, 300 nM ssDNA reporter; 4: 1 µM crRNA, 1 µM ssDNA reporter; 5: 1 µM crRNA, 2 µM ssDNA reporter; 6: 2 µM crRNA, 1 µM ssDNA reporter; 7: 5 µM crRNA, 1 µM ssDNA reporter; 8: 1 µM crRNA, 5 µM ssDNA reporter; 9: 10 µM crRNA, 5 µM ssDNA reporter; and 10: 10 µM crRNA, 10 µM ssDNA reporter. NC, Negative control (double-distilled H_2_O). **(A)** Fluorescence detection using a multifunctional microplate reader (λ_ex_: 485 nm, λ_em_: 520 nm). **(B)** Visible green fluorescence detection under a blue LED transilluminator at a wavelength of 470 nm. By measuring the fluorescence value, one-way ANOVA was performed on the fluorescence value with fluorescence and the fluorescence value of the negative control to obtain the result of P <0.0001. This indicates that the difference between fluorescence and non fluorescence is very significant, represented by "****".

**Figure 3:**
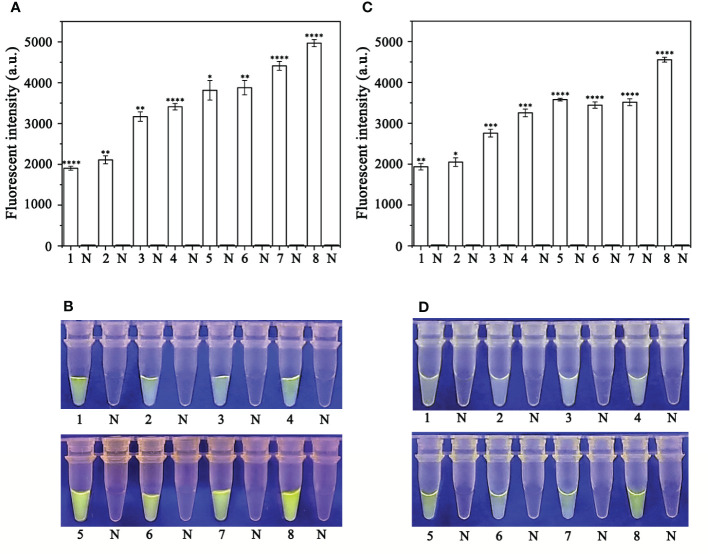
Optimization of the recombinase polymerase amplification (RPA) reaction time and Cas12a cleavage time for the RPA-CRISPR/Cas12a assay. **(A, B)** RPA reaction times: 1, 3, 5, 7, 9, 11, 13, and 15: 5, 10, 15, 20, 25, 30, 35, and 40 min; 2, 4, 6, 8, 10, 12, 14, and 16: NC (negative control, double-distilled H_2_O). **(C, D)** Cas12a cleavage times: 1, 3, 5, 7, 9, 11, 13, and 15: 5, 10, 15, 20, 25, 30, 35, and 40 min; 2, 4, 6, 8, 10, 12, 14, and 16: NC (negative control, double-distilled H_2_O). **(A, C)** Fluorescence detection using a multifunctional microplate reader (λ_ex_: 485 nm, λ_em_: 520 nm). **(B, D)** Visible green fluorescence detection under a blue LED transilluminator at a wavelength of 470 nm.

Various concentrations of crRNA and ssDNA reporter for the RPA-CRISPR/Cas12a assay were used to determine the optimal concentration (**Table S1**). The visible green fluorescence and fluorescence intensity results indicated that 10 μM crRNA and 10 μM ssDNA reporter were the optimal concentrations for the RPA-CRISPR/Cas12a assay (**Figures 2A, B**). Subsequently, 10 μM crRNA and 10 μM ssDNA reporter were used to test for the optimal RPA reaction time (assessed at the time points 5, 10, 15, 20, 25, 30, 35, and 40 min). Strong green fluorescence was observed with the blue LED transilluminator after 5 min and the fluorescence intensity increased from 5 min (**Figures 3A, B**). Therefore, the optimal RPA reaction time was 5 min. Assessment of the optimal Cas12a cleavage time (at 5, 10, 15, 20, 25, 30, 35, and 40 min) using the product of the 5 min RPA reaction revealed that the optimal cleavage time was 15 min (**Figures 3C, D**). In conclusion, green fluorescence was clearly visible after 20 min (with 5 min for the RPA reaction and 15 min for Cas12a cleavage) (**Figure 3**).

### Specificity of the RPA-CRISPR/Cas12a assay for rapid detection of *P. sojae*

**Figure 4:**
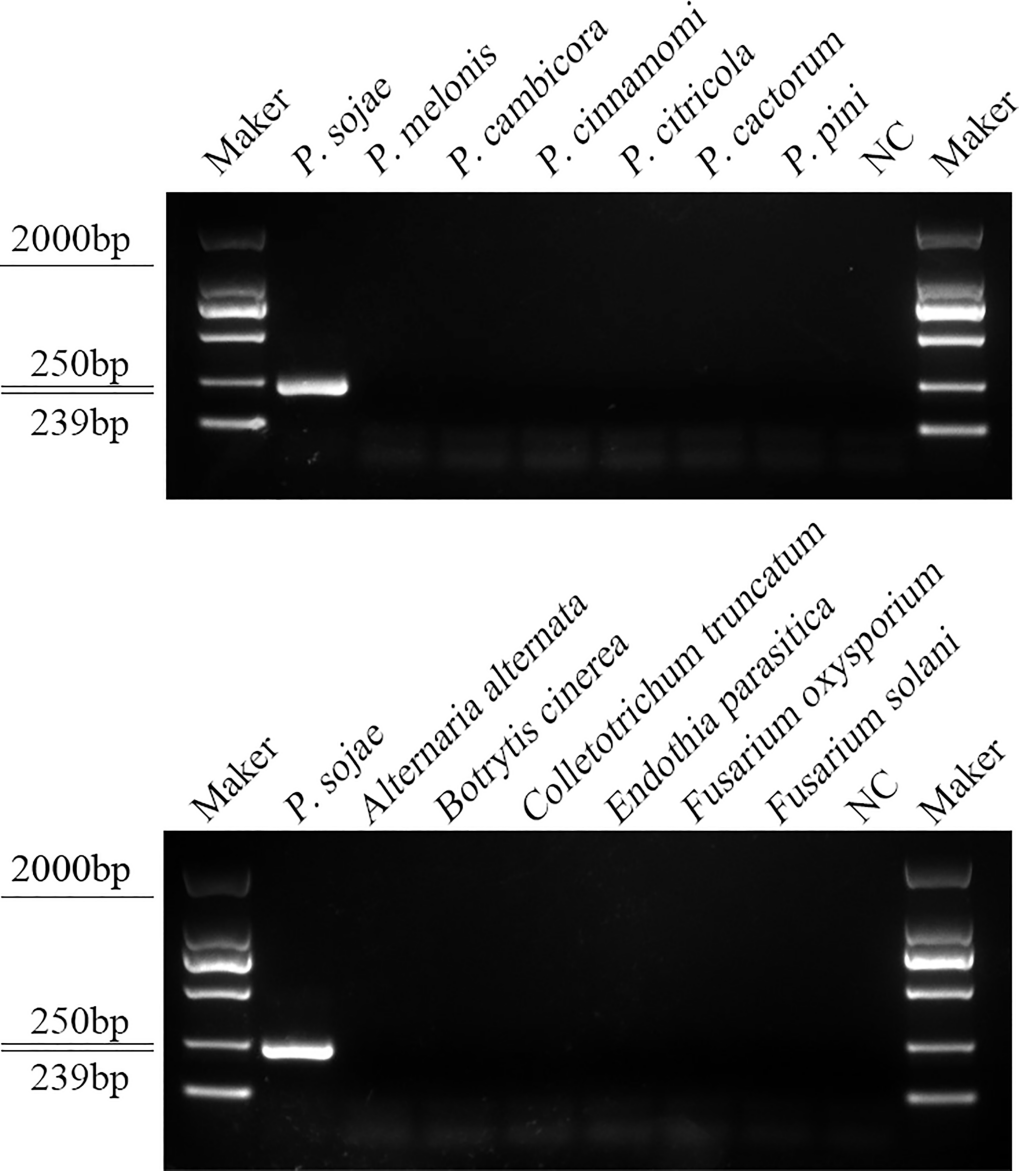
Primers designed based on the *Ypt1* gene sequence were screened for specificity using conventional PCR assays, and detected by 1.5% agarose gel electrophoresis. PCR amplicons (239 bp) were only detected in the *P. sojae* sample, indicating specificity for detection of *P. sojae* DNA. Marker DL2000 (Takara Shuzo, Shiga, Japan). NC, Negative control (double-distilled H_2_O).

**Figure 5:**
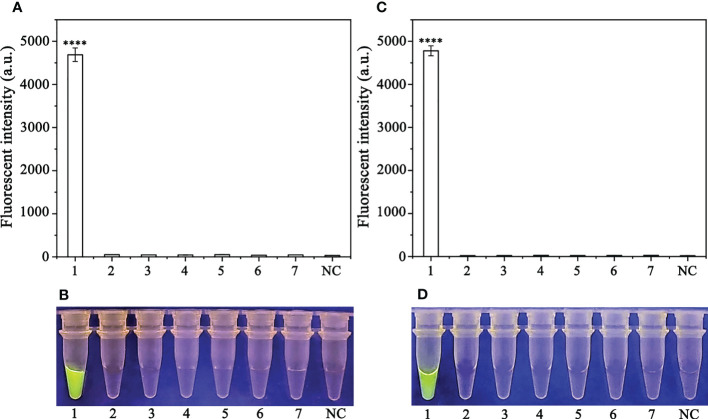
Evaluation of the specificity of the RPA-CRISPR/Cas12a assay. **(A, B)** Evaluation using genomic DNA isolated from 1: *Phytophthora sojae*, 2: *P. melonis*, 3: *P. cambivora*, 4: *P. cinnamomi*, 5: *P. citricola*, 6: *P. cactorum*, 7: *P. pini*, and 8: negative control (NC; no template). **(C, D)** Evaluation using genomic DNA from 1: *P. sojae*, 2: *Alternaria alternata*, 3: *Botrytis cinerea*, 4: *Colletotrichum truncatum*, 5: *Endothia parasitica*, 6: *Fusarium oxysporium*, 7: *F. solani*, and 8: negative control (NC; no template).

A PCR amplification product of approximately 239 bp was amplified in the RPA reaction from the gDNA of *P. sojae* with the primers Ypt1RPA-F and Ypt1RPA-R. No PCR amplicons were detected in the reactions with gDNA of *P. sansomeana*, *P. melonis*, *P. vignae*, *P. cinnamomi*, *P. cryptogea*, *P. citrophthora*, *Alternaria alternata*, *Botrytis cinerea*, *Colletotrichum truncatum*, *Endothia parasitica*, *Fusarium oxysporium*, *F. solani*, and the NC (**Figure 4**). In the specificity assessment for the RPA-CRISPR/Cas12a assay, a multifunctional microplate reader detected a strong fluorescence signal for gDNA of *P. sojae*, whereas no fluorescence signal was observed for gDNAs of other oomycetes and nematodes (**Figures 5B, D**). Green fluorescence from gDNA of *P. sojae* was clearly observed under a blue LED transilluminator at a wavelength of 470 nm, whereas no green fluorescence was detected for the gDNAs of other oomycete isolates, fungal isolates, and nematodes (**Figures 5A, C**). The specificity tests were replicated three times and each replicate yielded identical results.

### Sensitivity of the RPA-CRISPR/Cas12a assay for rapid detection of *P. sojae*

**Figure 6:**
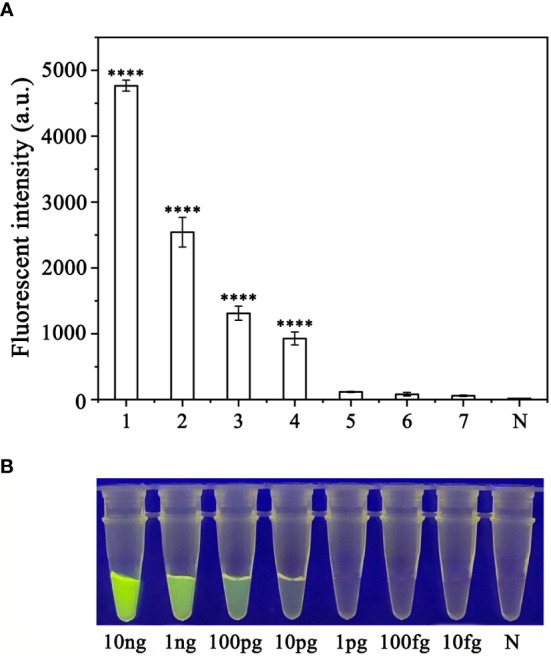
Evaluation of the sensitivity of the RPA-CRISPR/Cas12a assay for detection of *Phytophthora sojae*. The pathogen was detected at a minimum genomic DNA concentration of 10 pg µL^−1^ using **(A)** a multifunctional microplate reader (λ_ex_: 485 nm, λ_em_: 520 nm) or **(B)** a blue LED transilluminator at a wavelength of 470 nm. 1–8: 10 ng µL^−1^, 1 ng µL^−1^, 100 pg µL^−1^, 10 pg µL^−1^, 1 pg µL^−1^, 100 fg µL^−1^, 10 fg µL^−1^ and NC (negative control, double-distilled H_2_O). By measuring the fluorescence value, one-way ANOVA was performed on the fluorescence value with fluorescence and the fluorescence value of the negative control to obtain the result of P <0.0001. This indicates that the difference between fluorescence and non fluorescence is very significant, represented by "****".

To evaluate the sensitivity of the RPA-CRISPR/Cas12a assay for detection of *P. sojae*, 2 µL of *P. sojae* gDNA of various concentrations (10 ng.µL^−1^, 10 ng.µL^−1^, 1 ng.µL^−1^, 100 pg.µL^−1^, 10 pg.µL^−1^, 1 pg.µL^−1^, 100 fg.µL^−1^ and 10 fg.µL^−1^) were used. The gDNA concentrations of 10 ng.µL_−1_, 1 ng.µL_−1_, 100 pg.µL_−1_, and 10 pg.µL_−1_ resulted in visible green fluorescence, whereas the remaining gDNA concentrations and the NC did not produce visible fluorescence. All results were consistent among three replicates. These results indicated that the minimum detectable gDNA concentration was 10 pg.μL_−1_ gDNA (**Figure 6**). The RPA-CRISPR/Cas12a assay was repeated three times for each concentration of gDNA template under identical experimental conditions as described above.

### Detection of *P. sojae* in naturally infected soybean plants using the RPA-CRISPR/Cas12a assay

Based on combination of the simplified PEG-NaOH method and the RPA-

**Figure 7:**
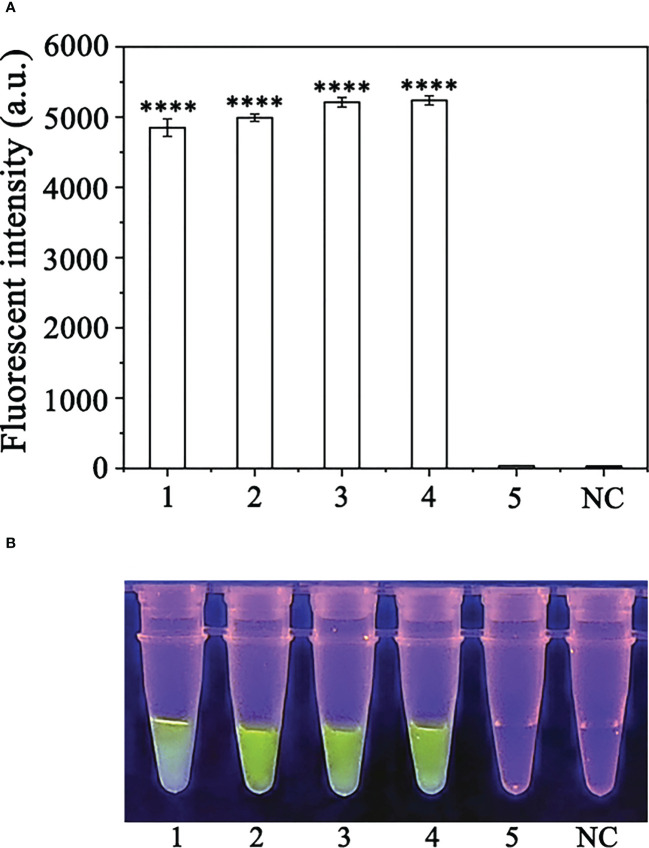
Detection *of Phytophthora sojae* in naturally infected soybean root samples using the RPA-CRISPR/Cas12a assay combined with a simple DNA extraction method. **(A)** Strong fluorescence signals were detected by a multifunctional microplate reader (λ_ex_: 485 nm, λ_em_: 520 nm). 1: Positive control, PC; 2-4: naturally infected soybean root samples; 5: healthy soybean root sample; NC: negative control. **(B)** Visible green fluorescence was detected under a blue LED transilluminator at a wavelength of 470 nm. 1-4: Naturally infected soybean root samples; 5: healthy soybean root sample; NC: negative control. By measuring the fluorescence value, one-way ANOVA was performed on the fluorescence value with fluorescence and the fluorescence value of the negative control to obtain the result of P <0.0001. This indicates that the difference between fluorescence and non fluorescence is very significant, represented by "****".

CRISPR/Cas12a assay, we detected *P. sojae* in naturally infected soybean samples. Using the RPA-CRISPR/Cas12a assay, green fluorescence was detected based on DNA extracted from the positive control and diseased samples, whereas no fluorescence was detected based on the DNA obtained from the NIS (non-inoculated samples) or the NC. All samples generated visible green fluorescence that was detected by a multifunctional microplate reader and yielded identical results (**Figures 7A, B**). These results demonstrated that the RPA-CRISPR/Cas12a assay combined with the simplified PEG-NaOH method could be used for effective detection of *P. sojae* in field-collected samples.

## Discussion

*P. sojae* is a destructive soilborne pathogen of soybean, present in most soybean-growing regions of the world, that can be difficult to diagnose and causes severe economic losses. The pathogen thrives in wet conditions and in compacted or heavy clay soils. Motile, water-borne zoospores were released from sporangia and were attracted to soybean root exudates (Morris and Ward, 1992). This pathogen can infect seeds, seedlings, and plants at all stages of growth when the soil conditions favor pathogen reproduction and infection. Symptoms are usually apparent 1–2 weeks after heavy rains and are most common where soils are poorly drained. Soybean root rot is among the most serious diseases in China. Accurate and rapid detection of *P. sojae* in plants and the soil is a critical step towards effective prevention and management of soybean root and crown rot and seedling damping-off.

Molecular detection technology is useful to rapidly monitor the incidence of *P. sojae*. *P. sojae* is easily mistaken for *P. megasperma*. Indeed, *P. sojae* and *P. medicaginis* were formerly known as *P. megasperma* f. sp. *glycinea* and *P. megasperma* f. sp. *medicaginis*, respectively. Molecular analysis of the mitochondrial region and isozyme analysis provided evidence that these were distinct species (Forster et al., 1989). Given the unique symptoms of soybean root rot in infected tissues, we aimed to establish a rapid detection technology to assist in early diagnosis of soybean root rot and the formulation of disease control strategies (Wang and Fang, 2005).

This is the first report of a RPA-CRISPR/Cas12a assay for rapid detection of *P. sojae*. The total amplification time was limited to 20 min (5 min for the RPA reaction and 15 min for the CRISPR/Cas12a assay) and the test results were visible under UV light and readout coming from fluorophores. The crRNA and ssDNA concentrations strongly influence the results of the RPA-CRISPR/Cas12a assay. Accordingly, different combinations of crRNA and ssDNA reporter concentrations were screened and the fluorescence intensity peaked with crRNA and ssDNA reporter concentrations of 10 μM and 10 μM, respectively. As demonstrated in the specificity evaluation, the present novel RPA-CRISPR/Cas12a system specifically detected gDNA of *P. sojae*, whereas no positive reactions to gDNA of 29 other *Phytophthora* species, including *P. medicaginis*, *P. megasperma*, *P. sansomeana*, *P. melonis*, and *P. vignae* (**Table 1**). This indicates that the method meets the requirements for specific detection of *P. sojae*. In addition, the lowest detectable gDNA concentration of *P. sojae* was 10 pg.µL^−1^ using the RPA-CRISPR/Cas12a assay. The feasibility of using this assay was investigated using soybean samples from a field infected with soybean root rot. The results demonstrated that the assay showed high accuracy, indicating its potential for early diagnosis of *P. sojae*.

This method has several advantages compared with conventional methods. First, the RPA-CRISPR/Cas12a reactions can be performed at a constant and relatively low temperature (37°C). Human body temperature, USB-powered holding boxes or thermostatic heaters can provide the required temperature conditions without the need for complex temperature control equipment, thereby eliminating a requirement for expensive specialized instruments, such as thermocyclers. Second, the entire diagnostic process can be completed within approximately 20 min. In contrast, a PCR-based detection method requires at least 2.5 h, including 90 min for the PCR reaction and 30 min for gel electrophoresis (Shin et al., 2021). Third, Some substances in the RPA reaction interfere with the antibodies on the test paper may causes non-specific binding and false positive signals to occur, which are not sufficiently diluted. Binding RPA to CRISPR-Cas12a allows us to detect the target twice, once for the recognition of the RPA primer and the second time for the recognition of the RPA amplification product by CRISPR-Cas12a when the RPA reaction is performed, which effectively avoids the problem of false positives during RPA amplification. Forth, it is not necessary to use high quality and purified DNA for amplification template which is required for conventional PCR-based diagnosis (Shin et al., 2021). That maked it an ideal choice for establishing a rapid on-site detection technology platform. A novel RPA-LFD assay was previously developed for accurate, simple, and rapid detection of *P. sojae* (Dai et al., 2019). The sensitivity of the RPA-CRISPR/Cas12a assay reported here is comparable, if not higher, than most previously developed detection methods. The detection limit for the RPA-CRISPR/Cas12a assay was 10 pg in a 50-μL reaction. Although the sensitivity was limited to 10 pg.μL^−1^, which is same with the RPA-LFD assay, the RPA-CRISPR/Cas12a assay sensitivity was sufficient to reliably detect *P. sojae.* The sensitivity is higher than that of a LAMP assay (Dai et al., 2012). Compared with the RPA-LFD assay, the present RPA-CRISPR/Cas12a detection system exhibits a number of promising advantages, including multiplexed detection capability, low requirement for sophisticated instruments and specialist personnel, and enhanced testing accuracy (Dai et al., 2019). Compared with the RPA-LFD method, the RPA-CRISPR/Cas12a technology enables isothermal label-free detection of target genes by designing RPA primers and performing isothermal amplification of the products obtained as amplification reporters to generate the CRISPR/Cas12a cleavage products has. Thus, the method offers the opportunity for development of an accurate, user-friendly, inexpensive platform for point-of-care testing application of CRISPR-based diagnostics.

This research aimed to develop a RPA-CRISPR/Cas12a assay for detection of *P. sojae.* The assay is easy to perform and portable, and does not require expensive equipment. Although these diverse properties of the CRISPR/Cas12a system provide potential for the development of versatile tools for pathogen detection (Kim et al., 2016a; Kim et al., 2016b; Li et al., 2018), there remain challenges to overcome, including few currently identified orthologs of Cas12a, limited genomic targeting coverage, and relatively low editing efficiency (Kleinstiver et al., 2016; Kim et al., 2017; Tu et al., 2017). In addition, different crRNA scaffolds affect the activities of the Cas12a–crRNA complex (Li et al., 2017). These scaffolds will help to elucidate the exact mechanisms of the reactions and enable improvements in the future. Meanwhile, the lyophilization application of CRISPR reaction needs to be further explored, and the reagents required for CRISPR reaction need to be lyophilized before use, which makes the application of CRISPR assay limited. The lyophilization application of the whole CRISPR reaction system has been reported (Nguyen et al., 2021; Rybnicky et al., 2022), and the lyophilization application of CRISPR reaction can be further explored in the future, so that the reagents can be stored for a long time at room temperature and really put into large-scale application. In addition, consecutive base changes and deletions were not investigated in this study, and there is still much room to explore whether RPA-CRISPR/Cas12a can recognize single-base differences.

The proposed CRISPR/Cas12a assay can be used for detection and identification of *P. sojae* in the field and laboratory. The RPA-CRISPR/Cas12a can complete the rapid detection of P. sojae at 37°C for 30 min.The accuracy of RPA-CRISPR/Cas12a was verified by collecting 30 soybean rhizosphere samples and has potentially significant applications to detect *P. sojae*. In-field diagnosis of soybean root rot would help to create an effective and accurate control strategy for farmers.

## Conclusions

The present novel RPA-CRISPR/Cas12a-based assay exhibits high specificity and sensitivity, and a short detection time. The test results were visible under UV light and readout coming from fluorophores (**Table S3**) without the need for expensive equipment and facilitates the early detection of *P. sojae*. The assay is sensitive, efficient, and convenient. Practitioners could consider improvements to this assay to increase the sensitivity and expand detection to other pathogens.

## Data availability statement

The original contributions presented in the study are included in the article/**Supplementary Material**. Further inquiries can be directed to the corresponding authors.

## Author contributions

YG conceptualized and designed the research, analyzed the data, interpreted the results, performed the experiments and wrote the manuscript. HX participated discussed the experimental design. TD and TL revised the manuscript and directed the project. All authors contributed to the article and approved the submitted version.
