# Supplementary figures and images for: RPA-CRISPR/Cas12a mediated isothermal amplification for visual detection of Phytophthora sojae
Source: Front Cell Infect Microbiol. 2023 May 26;13:1208837. doi: 10.3389/fcimb.2023.1208837 (PMC10250720; doi:10.3389/fcimb.2023.1208837)

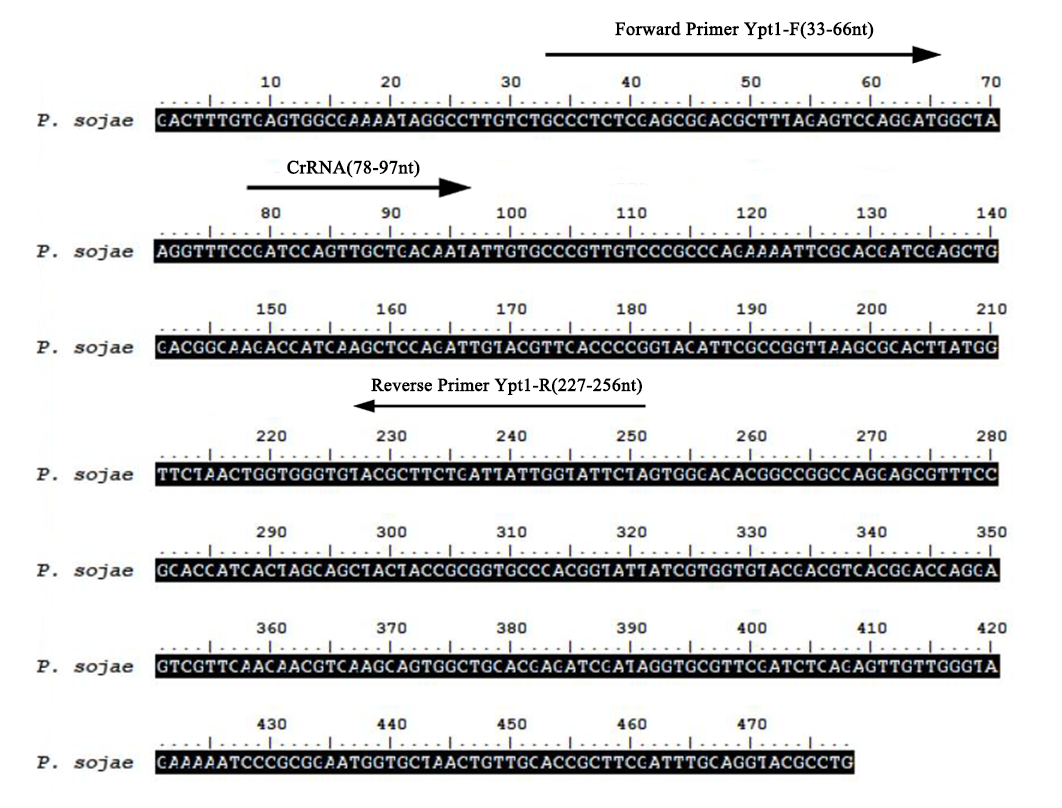

Supplement: Supplementary file 1 [file Image_1.tif]
